# Deficiencies in Planning Interventional Trial Registration of COVID-19 in China

**DOI:** 10.3389/fmed.2021.618185

**Published:** 2021-03-31

**Authors:** Xiaodan Li, Shengzhao Zhang, Yiling Zhou, Ying Liu, Youlian Zhou, Sheyu Li, Na Su

**Affiliations:** ^1^Department of Gastroenterology, West China Hospital, Sichuan University, Chengdu, China; ^2^Department of Pharmacy, West China Hospital, Sichuan University, Chengdu, China; ^3^Department of Endocrinology and Metabolism, West China Hospital, Sichuan University, Chengdu, China; ^4^Department of Guideline and Rapid Recommendation, Cochrane China Center, MAGIC China Center, Chinese Evidence-Based Medicine Center, West China Hospital, Sichuan University, Chengdu, China; ^5^West China School of Pharmacy, Sichuan University, Chengdu, China

**Keywords:** COVID-19, SARS-CoV-2, interventional trials, registries, China, sample size index

## Abstract

**Background:** The coronavirus disease 2019 (COVID-19) pandemic has affected the world since late 2019. The efforts to control the spread of the virus need to be supported by credible evidence. Therefore, we analyzed the rationality of the timeline and geographic distribution of COVID-19 trial registration in mainland China.

**Methods:** We searched the Chinese Clinical Trial Registry (ChiCTR, http://www.chictr.org.cn/) and International Clinical Trials Registry Platform (ICTRP, https://www.who.int/ictrp/en/) using keywords including novel coronavirus, coronavirus pneumonia, 2019-nCoV, COVID-19, and SARS-COV-2 from 1 December 2019 to 27 April 2020 and included interventional randomized and non-randomized trials including patients with confirmed cases of COVID-19 in mainland China. The registered trials were reviewed, and data were independently extracted by two reviewers based on the inclusion criteria.

**Results:** A total of 263 registered interventional trials were included in the study. We defined the sample size index (SI) as the total number of patients needed by the trials divided by the total number of patients diagnosed with COVID-19. A total of 84,341 patients had been diagnosed with COVID-19 in China as of 26 April 2020, and the included trials had a combined sample size of 31,156 patients (SI: 0.37). After control of the COVID-19 epidemic was achieved in China (February 18, 2020), the SI was 1.54, suggesting that the number of patients needed by the trials was greater than the number of newly diagnosed patients. The SIs in 8 out of 26 provinces in mainland China were >1.

**Conclusions:** Our results suggested a clear over registration of COVID-19 trials in China, especially after control of the pandemic was achieved, preventing the generation of high-quality evidence.

## Introduction

The coronavirus disease 2019 (COVID-19) pandemic has affected the world since late 2019 (1, 2). The efforts to control the spread of the virus need to be supported by credible evidence, and the performance of clinical trials and publication of the results substantially assist in the public health control and clinical management of the disease (3, 4). However, a waste of research resources has also been reported (5, 6). China was the first country to respond to the pandemic and published the earliest randomized controlled trials (RCTs) pertaining to COVID-19 (7–10). Since the number of newly diagnosed cases dramatically dropped in China starting in late February, many ongoing trials may face competition with other trials with regard to recruitment because of the limited number of patients. In addition, new trials pertaining to COVID-19 were still registered. In the current study, we analyze the rationality of the timeline and geographic distribution of COVID-19 trial registration in mainland China.

## Materials and Methods

### Data Source

We searched the Chinese Clinical Trial Registry (ChiCTR, http://www.chictr.org.cn/) and International Clinical Trials Registry Platform (ICTRP, https://www.who.int/ictrp/en/) using keywords including novel coronavirus, coronavirus pneumonia, 2019-nCoV, COVID-19, and SARS-COV-2 from 1 December 2019 to 27 April 2020 and included interventional randomized and non-randomized trials that included patients with confirmed cases of COVID-19 in mainland China. Registered trials were included if they: (1) involved patients with confirmed cases of COVID-19; (2) were interventional clinical trials; (3) were single-arm or controlled; and (4) were conducted in mainland China.

Two authors (XL, SZ) obtained the number of patients in each province from the National Health Commission of the People's Republic of China (http://www.nhc.gov.cn/).

### Data Extraction

Two authors (XL, SZ) independently extracted the number of confirmed cases with COVID-19 in each province per day and the following data of included clinical trials, registration number, date of registration, public title, applicant's institution, ethical approval, study type, study design, study phase, inclusion criteria, exclusion criteria, randomization, blinding, study period, funding, interventions, sample size, research setting, main outcomes, and surrogate outcomes including adverse events, imageological examination, laboratory examination, viral nucleic acid, pulmonary function, measuring scale assessing and recovery time/length of stay. These extracted data were cross-checked, and any discrepancies were resolved through discussion or negotiation with a third party.

The trial location was defined as the province where patients were recruited. For a multicenter trial which did not report exact number or percentage of participants from each province, its sample size would be evenly distributed among these provinces by one author (YZ).

### Data Analyses

The data were double-entered and cross-checked using Excel 2019 for the preliminary extraction of the data pertaining to the registered trials. We summarized the number of newly confirmed cases per day, the cumulative number of confirmed cases, sample size of newly registered trials per day, and the cumulative sample size of registered trials from 1 December 2019 to 27 April 2020. We calculated sample size index (SI) using the total number of patients needed by the trials divided by the total number of patients diagnosed with COVID-19. An SI >1 suggests some trials could not be completed unless some patients were involved in more than one trial. We divided the data into two groups using February 18 as the cut-off point. We generated heat maps and time diagrams in R Studio (version 3.6.1).

## Results

A total of 721 registered studies were retrieved, including 625 from ChiCTR and 96 from the WHO ICTRP. After screening the registration information, 263 registered trials with a total sample size of 31,156 participants were included in the final analysis. The reasons for the exclusions were as follows: (1) epidemiologic studies (*n* = 125); (2) not related to COVID-19 (*n* = 70); (3) revocation of authorization (*n* = 52); (4) not interventional studies (*n* = 172); (5) discharged patients (*n* = 34); (6) duplicate registration (*n* = 5). A total of 84,341 patients had been diagnosed with COVID-19 by 26 April 2020, and the overall SI was 0.37. This suggests that every one in three patients with COVID-19 needed to participate in a trial to facilitate the completion of all trials.

### Baseline Characteristics of Registered Trials

The registered study locations were distributed across 26 provinces and municipalities in mainland China, and 26 were multicentre clinical studies. The first trial was registered on 23 January 2020. There were 183 RCTs (69.6%), 38 non-randomized controlled trials (14.4%), and 42 single-arm trials (16.0%). Only 191 registered trials (72.6%) obtained ethical approval, and 38 (20.8% among the RCTs) used blinding. The median sample size was 78 (range: 4 to 1,000). There were 240 (91.3%) principle investigators who belonged to medical institutions. As shown in Table 1, the interventions included drug therapy (138, 52.5%), non-drug therapy (47, 17.9%) and alternative therapy (78, 29.7%). Alternative therapy including Chinese traditional medicine (72, 27.4%), non-drug therapy in Chinese traditional medicine (5, 1.9%) and other alternative therapy (1, 0.4%). The outcomes included patient-important outcomes (70, 26.6%) and surrogate outcomes (193, 73.4%).

**Table 1:** The baseline of the included registered trials.

		**Number (*n*)**
The number of registered trials		263
Intervention	Drug therapy	138
	Non-drug therapy	47
	Alternative therapy	78
	Chinese Traditional Medicine	72
	Non-drug therapy in Chinese Traditional Medicine	5
	Other alternative therapy	1
Outcome	Patient-important outcome(s)	70
	Surrogate outcome(s)	193

*Drug therapy: Drugs for gastrointestinal disorders; Drugs for blood and blood forming organs; Drugs for cardiovascular; Hormone; Anti-infectious drugs; Antitumor and immune drugs; Nervous system drugs; Antiparasitic drugs; Respiratory system durgs; Other drugs; A combination of the two drugs (western medicine); Triple therapy (western medicine)*.

*Non-drug therapy: Respiratory support; Respiratory rehabilitation training; Dialysis treatment; nutrition support; Psychological therapy; Microecology/Light therapy/Mongolian medicine; Stem cell therapy*.

*Chinese Traditional Medicine: Traditional Chinese Medicine decoction pieces; Chinese Traditional medicine; Traditional Chinese medicine injection; Combination of Chinese and western medicine*.

*Non-drug therapy in Chinese Traditional Medicine: Acupoint treatment; Others*.

*Other alternative therapy: probiotics*.

*Patient-important outcome(s): Death or cure; Cure rate*.

*Surrogate outcome(s): Adverse outcomes; Imageological examination; Laboratory examination; Viral Nucleic Acid; Pulmonary function; Measuring scale assessing; Recovery time/length of stay*.

### Over Registration of Trials Based on the Timeline

**Figure 1:**
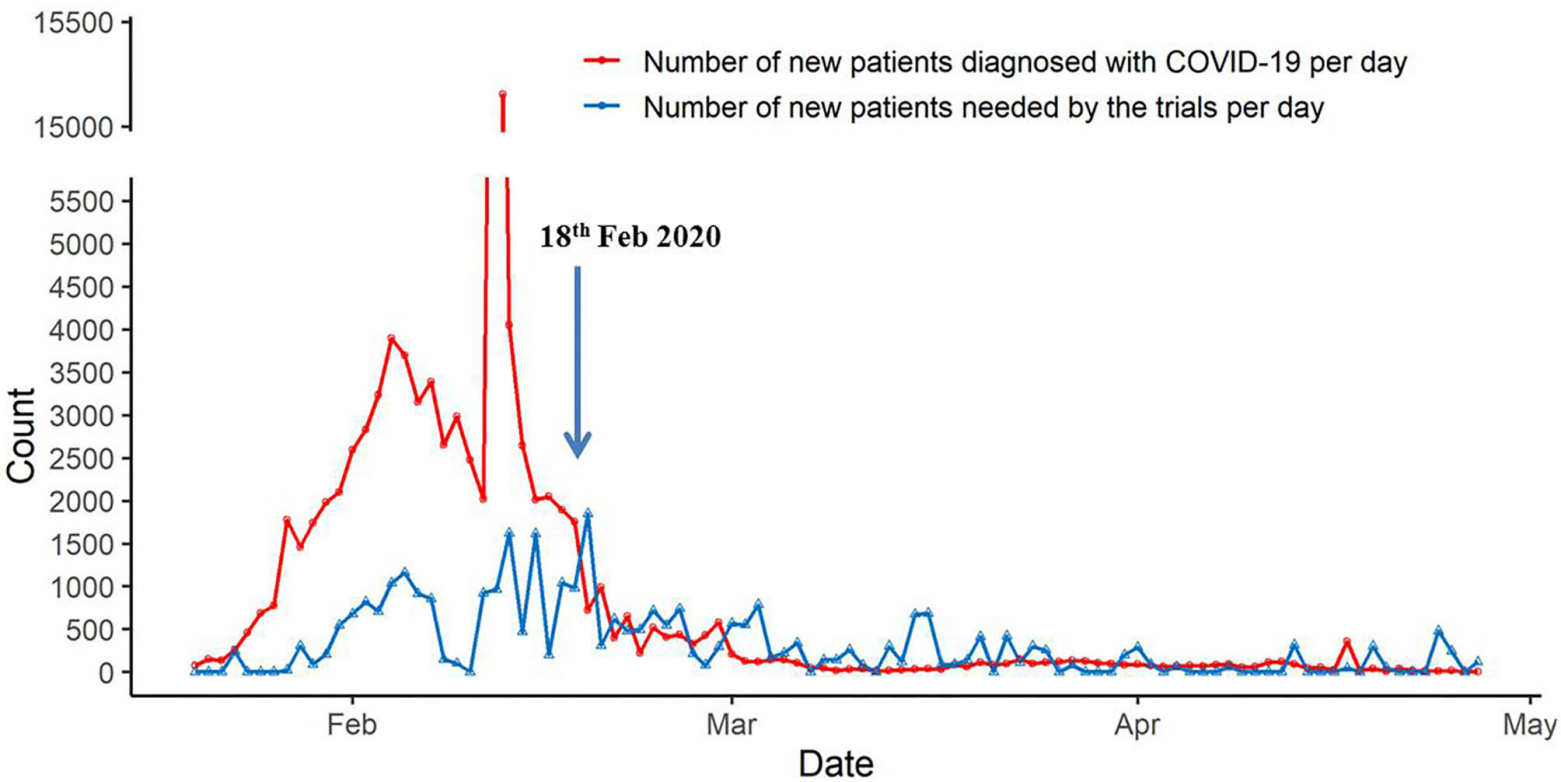
The timeline of new sample size needed by the trials and the new cases with COVID-19.

As shown in Figure 1, the number of new patients diagnosed with COVID-19 per day was greater than the number of new patients needed by the trials per day during the COVID-19 outbreak until February 18, which made recruitment feasible. On February 18, 982 new patients were needed by the trials, and there were 1,751 new patients diagnosed with COVID-19 (SI: 0.56), but on February 19, 1850 new patients were needed by the trials, and 723 new patients were diagnosed with COVID-19 (SI: 2.56). From starting on February 19 until 26 April, the number of new patients diagnosed with COVID-19 per day and the number of new patients needed by the trials per day fluctuated slightly, and on April 26, the number of new patients needed by the trials per day was greater than the number of new patients diagnosed with COVID-19 per day.

**Figure 2:**
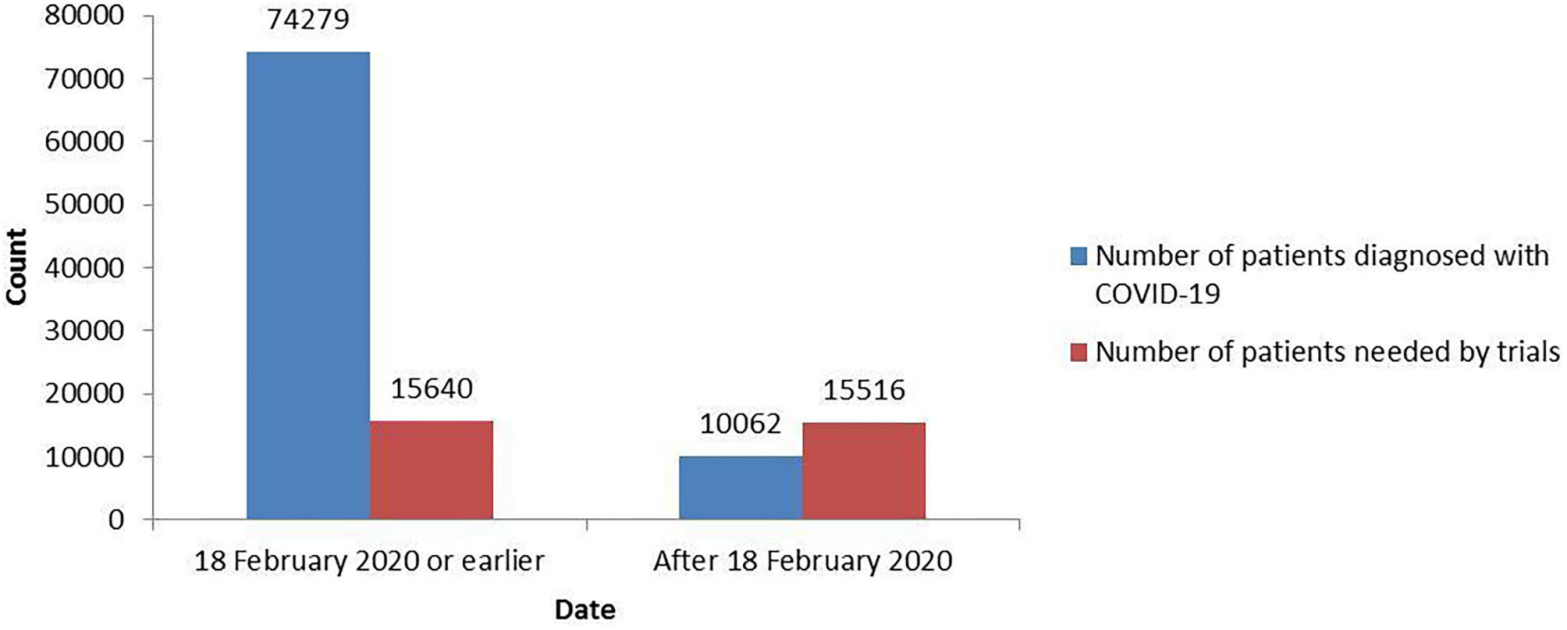
The total number of patients needed by the COVID-19 trials and the total number of patients diagnosed with COVID-19 before and after 18 February 2020.

As shown in Figure 2, 74,279 patients had been diagnosed with COVID-19 as of 18 February 2020 in China, at which point the registered trials needed to include 15,640 patients (SI: 0.21). After 18 February 2020, only 10,062 patients were newly diagnosed with COVID-19, but the inclusion of 15,516 new patients (SI: 1.54) was planned by the registered trials. The trials registered after 18 February 2020 needed too many patients to complete, and these trials may not be feasible.

### Over Registration of Trials by Location

**Figure 3:**
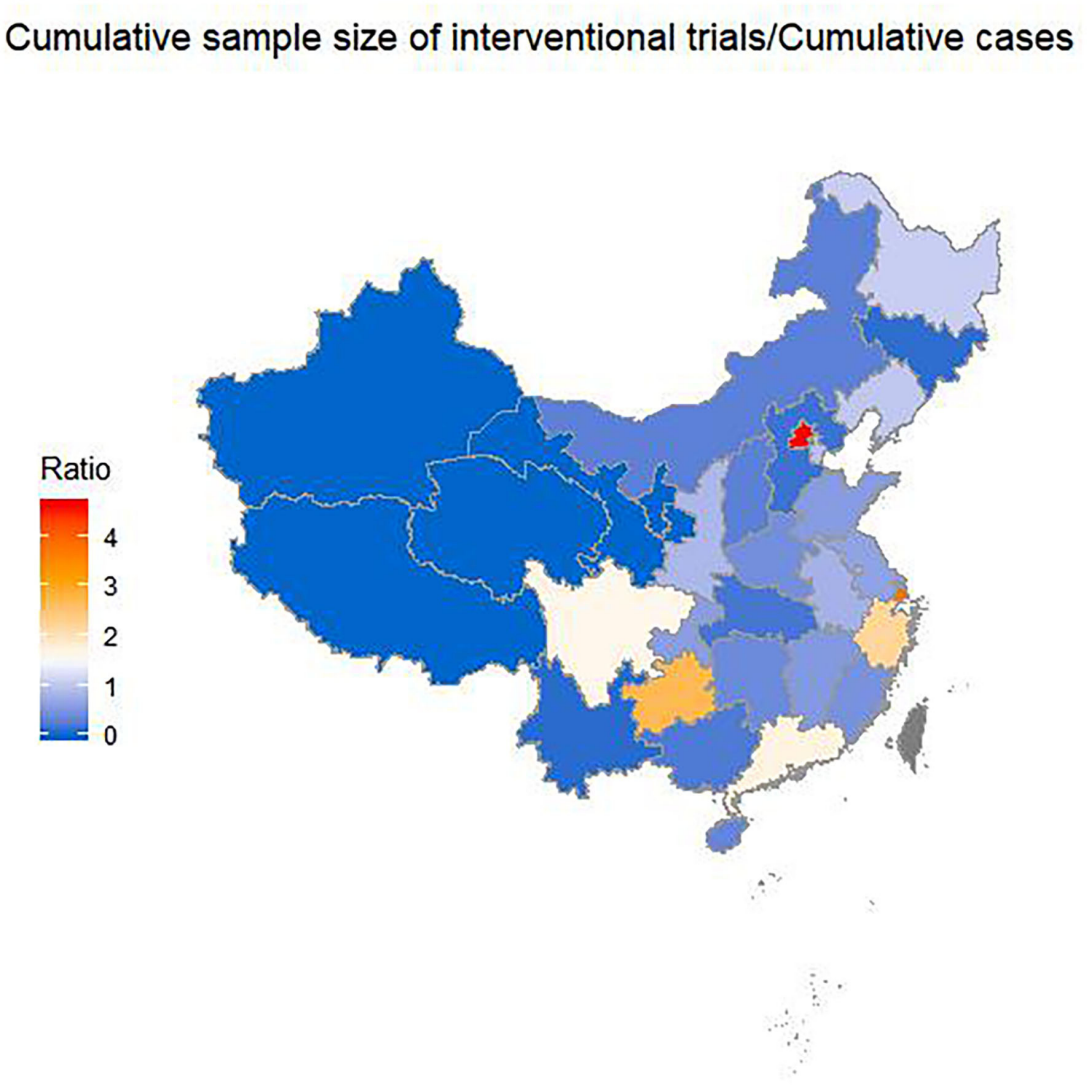
Sample index across provinces by 26 April 2020. Sample index is the ratio of the cumulative sample size needed by the trials to the cumulative confirmed cases with COVID-19.

Among the 26 provinces with registered trials in mainland China, the SIs of eight provinces were >1 (Figure 3). The top-five provinces were Beijing (SI: 4.64), Shanghai (SI: 3.85), Guizhou (SI: 2.72), Zhejiang (SI: 2.24), and Guangdong (SI: 1.74). All of these provinces except Guizhou Province are in East China. This means that the patients in Beijing would have to participate in an average of 4.64 trials to facilitate the completion of all the trials. The SI was 0.2 in Hubei Province, of which the cumulative confirmed cases represented 82.3% of total ones in China as of 26 April in China.

## Discussion

Our study showed that the timeline and geographic distribution of the COVID-19 trial registration in mainland China were unreasonable. The imbalanced distribution of the trial registration may be associated with the capacity for medical research across provinces. Novel therapies are urgently needed to address the COVID-19 crisis; however, our results suggest that many trials registered after 18 February, and those registered in the provinces with SIs >1 may not be completed because of competition for participants. This over-registration leads to the failure of trials. Many trials have had to terminate after recruiting a small number of patients before they could generate credible conclusions. Well-designed multicentre trials addressing important questions changed current practices relatively more efficiently (11), especially given the limited number of patients.

According to the basic information of the registered clinical trials included in this study, only 2/3 of the trials were randomized, and 20% of them performed blinding. The minimum sample size of the included registered studies was only 4, which is inadequate, making it difficult to produce meaningful conclusions. This suggests that the overall quality of the designs of these trials needs improvement, which is consistent with previous reports (12, 13). The median sample size of each trial was limited, but the overall sample size was large due to the fact that there were many trials. Over-registration may lead to excess trials, leading to the overexploitation of research sources by underpowered trials.

Over-registration may be caused by lacking of collaborations between researchers and institutes and the imbalanced distribution of medical services and research resources. The hospitals treating most patients with COVID-19 may not have the capacity to plan and conduct the trials. The feasibility of these trials thus may not be adequately considered, including the relevance of the topic, the appropriateness of the study design, and the calculation of the sample size. As there is no feedback system auditing trials without results, investigators may not adequately consider the consequences of the premature suspension of the trial on the availability of adequate financial support for COVID-19 research. A credit system to audit these prematurely suspended trials (e.g., not allowing investigators of these trials to register again in the system) may also help reduce over-registration.

Based on our analysis, the proportion of the surrogate outcome was too high. The registered studies used surrogate outcomes as the study endpoints instead of patient-important outcomes, which is a serious violation of the original intent of the registration of clinical trials involving patients with both mild and severe disease, making it impossible to determine the effectiveness of the interventions even if the studies are successfully performed. In addition, although Chinese traditional medicine therapy is important in China, it is over-represented in the registered trials. Instead of focusing on Chinese traditional medicine, we should perform more clinical trials on antiviral drugs, such as chloroquine and lopinavir/ritonavir.

Paul P. Glazious et al., in an editorial in the BMJ (14), pointed out that the number of COVID-related registered trials is large, but too many of them have small sample sizes, inaccurate designs and duplicate aims, which not only limits the possible benefits of the research but also wastes a substantial amount of resources. Sanders et al.'s review in JAMA (15) concluded that the number of relevant trials is growing rapidly, but there is still a substantial lack of RCTs among the currently registered studies, and to date, no effective drug treatment has been identified. The timely registration of trials should culminate in the publication of study results; however, the results thus far published have limited clinical implications. It should also be noted that the indirectness between clinical trials and real-world practice (16). Observational studies based on real-world data are equally necessary.

## Conclusion

Our study shows a clear over-registration of trials pertaining to COVID-19 in China, especially after control of the pandemic was achieved. We call for the proper regulation of trial registration and broader collaboration before designing a trial, especially during this public health crisis in China and other countries. A credit system to prevent the investigators of prematurely terminated trials from registering another study may be necessary in the future.

## Data Availability Statement

The original contributions presented in the study are included in the article/supplementary material, further inquiries can be directed to the corresponding author/s.

## Author Contributions

XL, SL, and NS: conceiving this study. XL, SZ, and YoZ: collecting the data. YL and YiZ: performing the analyses and illustrated the figures. XL, YL, SL, and NS: writing draft. All authors contributed to the article and approved the submitted version.

## Conflict of Interest

The authors declare that the research was conducted in the absence of any commercial or financial relationships that could be construed as a potential conflict of interest.
